# Carbon footprint for farms in the Czech Republic: a benchmark‐based assessment

**DOI:** 10.1002/jsfa.14368

**Published:** 2025-05-12

**Authors:** Jan Kovanda, Svatava Janoušková, Tomáš Hák, Viktor Třebický, Petr Koňata

**Affiliations:** ^1^ Charles University Environment Centre Prague 6 Czech Republic; ^2^ Faculty of Science Charles University Prague 2 Czech Republic; ^3^ Faculty of Humanities Charles University Prague 8 Czech Republic; ^4^ CI3, s.r.o Prague 2 Czech Republic; ^5^ CleverFarm, a.s Brno Czech Republic

**Keywords:** carbon footprint (CF), GHG emissions, agricultural farms, benchmarking, Czech Republic

## Abstract

**BACKGROUND:**

Climate change is a pressing environmental and social challenge that demands effective monitoring of greenhouse gas (GHG) emissions. One widely adopted approach for this is quantifying the carbon footprint (CF). Given that agriculture is a major contributor to GHG emissions, we have developed a comprehensive framework for CF accounting at the farm level. This framework has been tested on 12 farms in the Czech Republic to assess both data availability and calculation accuracy.

**RESULTS:**

Our study examines how various farm characteristics, such as turnover, land area and number of employees, influence the overall CF and enable meaningful comparisons between farms. We found that absolute farm CFs are significantly influenced by the size effect, making them unsuitable for benchmarking purposes. By contrast, relative farm CFs (per turnover, per area and per employee) are not affected by the size effect, but can be affected by a scale effect. Additionally, we investigated whether a focus on animal husbandry leads to higher relative CFs. By calculating the share of animal husbandry (SoAH) in farm operations, we discovered a significant correlation between SoAH and relative CFs, with the strongest correlation observed for CF per turnover (0.87).

**CONCLUSION:**

We argue that farms with high shares of SoAH are unlikely to reduce their relative CFs to the levels of farms with zero or low SoAH. We therefore propose applying benchmarking to farms with similar SoAH. We also propose that further research should focus on defining and validating relevant reference values, comprising a benchmark set that reflects different farm types. © 2025 The Author(s). *Journal of the Science of Food and Agriculture* published by John Wiley & Sons Ltd on behalf of Society of Chemical Industry.

## INTRODUCTION

Climate change is a critical environmental and social challenge discussed among experts, policymakers, civil society organizations, media and the public. The accumulation of greenhouse gases (GHG) from fossil fuel combustion and land‐use changes during the Anthropocene has led to an unprecedented rise in the Earth's average temperature.^1^ This has triggered global environmental shifts, including ocean acidification, sea‐level rise, extreme weather events, biodiversity loss and disruptions in biogeochemical cycles.^2^ Scientific evidence confirms these transformations, with some even visible to policymakers and the public. Consequently, there is increasing recognition that tackling this crisis requires rapid transitions in energy, land use, urban planning, critical infrastructure and industry.^3^

Effective mitigation and adaptation strategies rely on monitoring, particularly GHG emissions. Over the past decade, carbon footprint (CF) accounting has become a key tool for measuring emissions, often representing carbon dioxide or other greenhouse gases in carbon dioxide equivalents.^4^ As a result, various initiatives, guidelines and methodologies have emerged to quantify corporate‐level direct and indirect emissions.^5^ Given agriculture's significant GHG contribution, pressure has grown to implement reduction measures on farms. Researchers and farmers have responded by focusing on CF calculations to track emissions and evaluate mitigation strategies.^6^, ^7^, ^8^

Although the Intergovernmental Panel on Climate Change (IPCC) Guidelines standardize direct emissions from specific farm processes,^9^ a unified and accredited CF framework for farms is still lacking.^10^, ^11^ The present study proposes a conceptual CF accounting framework rooted in agricultural emissions theory, encompassing all emission types discussed in the literature. The framework and its calculations were tested on 12 Czech farms in 2023 to assess robustness and explore how farm characteristics and other factors influence CF results. Additionally, we examined result interpretations, emphasizing benchmarking to provide valuable insights for farms and stakeholders such as banks, investors and consumers.

## MATERIALS AND METHODS

### The farm CF overall framework

**Figure 1:**
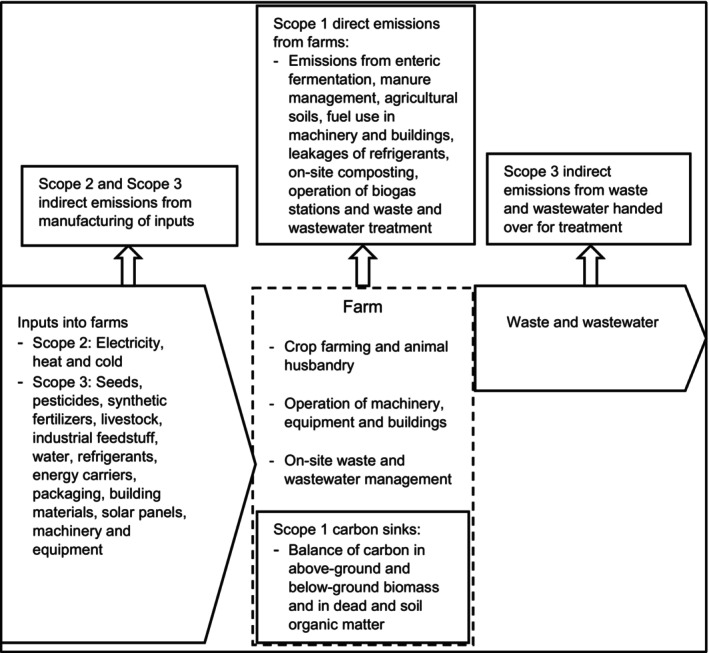
Framework for carbon footprint accounting on a farm level. Farm boundaries are indicated by a dashed line.

Figure 1 presents the conceptual framework for CF accounting at the farm level, designed to fully align with the GHG Protocol Agricultural Guidance.^12^ The GHG Protocol is a global partnership involving businesses, non‐governmental organizations, governments and other stakeholders. Its mission is to establish and promote widely accepted best practices for measuring and reporting greenhouse gas emissions.

The GHG Protocol and our framework classify GHG emissions into three main categories:

- Scope 1: Direct emissions and carbon sinks from farm activities
- Scope 2: Indirect emissions from electricity, heat and cold consumed on the farms
- Scope 3: Indirect emissions from manufacturing farm inputs, as well as emissions from waste and wastewater sent for treatment

The total CF at the farm level is calculated using the formula:

CF at the farm level = Scope 1 direct emissions + Scope 2 and Scope 3 indirect emissions from input manufacturing + Scope 3 indirect emissions from waste and wastewater – Scope 1 carbon sinks.

As a result of data limitations, our calculation does not include emissions related to farm workers' needs, such as transportation to work or food consumption. The GHGs considered in the analysis include carbon dioxide (CO_2_), methane (CH_4_) and nitrous oxide (N_2_O), which were converted into carbon dioxide equivalents (CO_2eq_) using global warming potentials (GWPs) of 1 for CO_2_, 28 for CH₄ and 265 for N_2_O. We also accounted for refrigerant leakages, applying their specific GWPs.^13^

To ensure the framework's completeness and the accuracy of emission quantification procedures, experts involved in the National Greenhouse Gas Inventory Reports, specifically Chapter 5 on Agriculture, validated the scheme presented in Figure 1.^13^

### Scope 1 direct emissions and carbon sinks

Scope 1 direct emissions and carbon sinks occur within the farm's boundaries. These are areas directly managed by farm administrators (owners or lessees). We categorize these GHG flows into four types:

1. Direct emissions from crop farming and animal husbandry
2. Direct emissions from machinery, equipment and building operations
3. Direct emissions from on‐site waste and wastewater management
4. Carbon sinks in soils

The first category is the most diverse. It includes CH₄ emissions from enteric fermentation, CH_4_ and N_2_O emissions from manure management and N_2_O emissions from agricultural soils. These emissions result from applying synthetic N fertilizers, manure, sewage sludge, digestate from biogas stations, compost, N‐crop residues, and animal urine and dung. We used standardized equations from the IPCC Guidelines for National Greenhouse Gas Inventories, particularly Volume 4 on Agriculture, Forestry and Other Land Use.^9^ Coefficients from the Czech Republic's National Greenhouse Gas Inventory Report^13^ were also applied. Farms provided data on livestock quantities, manure management systems (e.g. anaerobic digesters, liquid storage, solid storage, daily spread, pasture spread) and nitrogen application on soils.

The second category includes emissions from burning fuels for machinery, equipment and buildings, as well as refrigerant leakages from cooling systems. We used fuel consumption data from farms and emission coefficients for CO_2_, CH_4_ and N_2_O per unit of fuel burned. These coefficients were sourced from the UK Departments for Energy Security and Net Zero, and for Environment, Food & Rural Affairs.^14^ We calculated refrigerant leakages based on the GWPs of specific refrigerants.^13^ Farms provided data on fuel consumption, broken down by type (coal, natural gas, petrol, diesel, biomass) and refrigerant use in cooling systems.

The third category includes CH_4_ and N_2_O emissions from composting, biogas operations and wastewater treatment on farms. Although composting and biogas operations are common, none of the tested farms had wastewater treatment plants. Emissions from incinerators and landfills were excluded because it is unlikely that any farm operates such facilities. We calculated emissions from composting, biogas stations and wastewater treatment using coefficients from the Czech National Greenhouse Gas Inventory Report^13^ and farm data on composted waste, methane production in biogas stations and treated wastewater (measured by chemical oxygen demand, i.e. COD). For biogas stations, emissions were considered zero if the farm had a valid certificate ensuring no methane leakage. Otherwise, we assumed a 5% leakage rate, as recommended by the IPCC.^9^

The fourth category involves carbon sinks. These are calculated as the balance of carbon in above‐ground and below‐ground biomass and in dead and soil organic matter that lasts longer than a year. We calculated this for common farm land uses such as cropland and grassland, as well as land‐use conversions between these and forests (forest land is excluded). We distinguished two types of cropland: standard tillage and no‐tillage management. Carbon sink coefficients for cropland, grassland and land‐use conversions were taken from the Czech National Greenhouse Gas Inventory Report.^13^ For cropland under no‐tillage management, we sourced the carbon sink coefficient from Haddaway *et al*.^15^

### Scope 2 and Scope 3 indirect emissions

Scope 2 indirect emissions occur outside the farm boundaries during the production of electricity, heat and cold (e.g. ice for cooling) purchased by the farm. These emissions were calculated using emission coefficients for CO_2_, CH_4_ and N_2_O per unit of consumed electricity, heat, and cold, sourced from the Ecoinvent database.^16^

Scope 3 indirect emissions also occur outside the farm and include emissions from the production of purchased farm inputs and emissions from waste and wastewater sent for treatment. Purchased farm inputs were categorized into several groups: crop farming inputs (seeds, pesticides, synthetic fertilizers), animal husbandry inputs (livestock, industrial feed, silage, hay), water, refrigerants, energy carriers (e.g. coal, natural gas, petrol, diesel, biomass), packaging (plastic, paper), building materials (concrete, plasterboard, ceramic, iron, aluminum), solar panels and machinery/equipment (tractors, harvesters, tillage machines, trucks, cars, computers, etc.). Waste handed over for treatment was categorized as paper, plastic, metal, bio, glass, construction, municipal and other waste; wastewater was quantified as COD.

Emissions from the production of inputs and waste treatment were calculated using coefficients from the Ecoinvent database,^16^ whereas wastewater emissions used the same coefficients as on‐site wastewater treatment. Emissions from manufacturing farm inputs were attributed to the year the input was purchased. For solar panels and machinery, we adjusted the emissions over their entire lifespan, dividing the coefficients by the number of years of use. The data for these items were based on the total stock of solar panels and machinery, not just annual purchases, to account for previous years' investments. This approach helped smooth out emissions spikes on smaller farms that invest in such items infrequently.

### Data collection for the pilot calculation

A methodology for CF accounting on a farm level was tested in 12 volunteering farms. Table 1 shows basic facts about these farms ordered by their turnover.

**Table 1:** Basic facts about the tested farms, Czech Republic, 2022

Farm no.	Turnover (thousand EUR)	Area (ha)	Number of employees	Farm production for sale
1	24.4	24	1	Fruit, mutton meat
2	577.4	356	9	Cow milk
3	724.6	378	5	Cereals, pork meat
4	958.9	176	12	Grapes
5	1834.7	1673	28	Cereals, cow milk
6	3256.7	1295	9	Cereals, soya, rapeseed, sugar beet
7	4463.7	1208	21	Cereals, rapeseed, hop, cow milk
8	4694.5	1391	22	Cereals, sunflower seed, beef meet, cow milk
9	6839.0	1844	55	Cereals, rapeseed, poppy seed, beef meat, cow milk
10	8859.2	2252	66	Cereals, rapeseed, poppy seed, beef meat, cow milk
11	9191.7	3528	58	Cereals, rapeseed, beef meat, cow milk
12	33 368.7	5024	156	Cereals, rapeseed, poppy seed, vegetables, beef meat, pork meat

Note: Czech crowns were converted to EUR using the exchange rate of the Czech National Bank for 2022.^17^

The testing farms are a varied sample of agricultural enterprises; they differ in all size‐related characteristics, comprising turnover, area and number of employees, by several orders of magnitude. Also, the farms' focus is quite heterogeneous, from pure crop farming to pure animal husbandry with several inter‐forms.

Annual turnover is an important indicator showing how well the farm is performing each year. It usually refers to the total income made by a business over a year, also called ‘gross revenue’ or ‘total sales’. In terms of the area, a farm may have various definitions. It is typically a piece of land that has been cleared of its natural vegetation and is used to grow crops or keep animals. It also refers to the buildings used for keeping animals, storage of machinery, fertilizers and seeds, etc., and for farm administration. For the present study, we calculated the farm area as a sum of cropland, grassland (pastures and hayfields) and a built‐up area. Finally, employees refer to all professions required to keep farms running. These comprise farm owners and managers and farmworkers (milkers, tractor drivers, machinery operators, etc.).

We visited all tested farms in 2023 to distribute and explain the data form (Excel; Microsoft Corp., Redmond, WA, USA) detailing the required data for the CF calculation (see Supporting information, Data S1). The farms were asked to complete the data form for the last available year (i.e. 2022). The project team was available for further consultations, and the data collection was finalized at the end of 2023. The CF for all 12 farms was then calculated using the above‐described methodology.

## RESULTS

**Figure 2:**
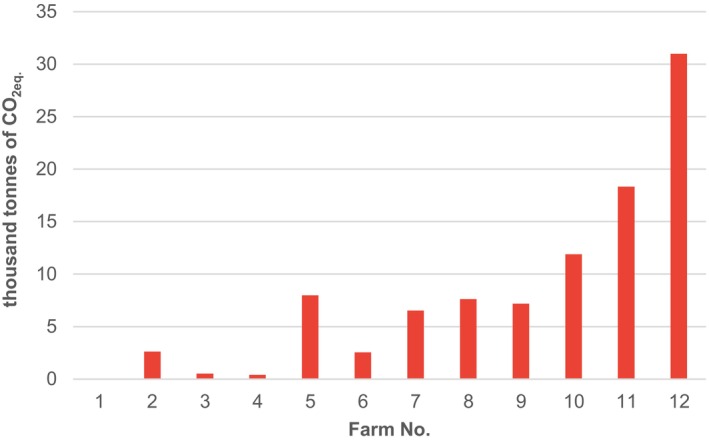
Absolute CFs for 12 tested farms, Czech Republic, 2022.

Figure 2 shows the absolute CFs calculated for the 12 tested farms.

The absolute CFs vary by three orders of magnitude—from roughly 49 tonnes (Farm 1) to 31 000 tonnes of CO_2eq_ (Farm 12).

**Figure 3:**
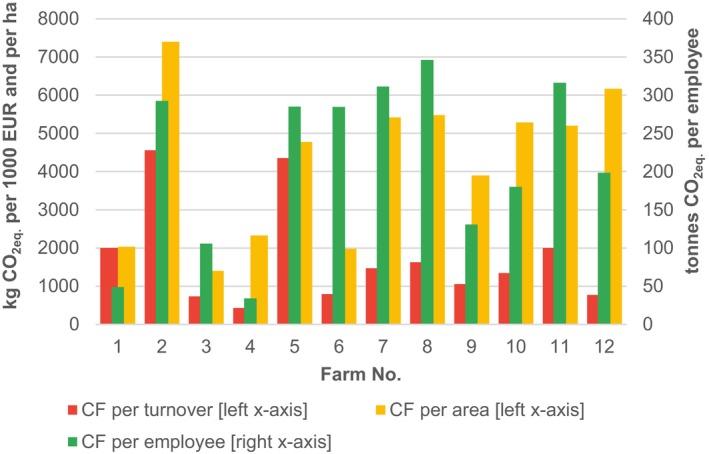
Relative CFs per turnover, per area and per employee, for 12 tested farms, Czech Republic, 2022.

Figure 3 shows relative CFs per turnover, per farm area and per employee.

Differences in relative CFs are much lower compared to absolute CFs, but they are still significant: CFs per turnover vary from 427 kg (Farm 4) to 4557 kg of CO_2eq_ per 1000 EUR (Farm 2), CFs per area vary from 1399 kg (Farm 3) to 7397 kg of CO_2eq_ per ha (Farm 2) and CFs per employee from 34 tonnes (Farm 4) to 346 tonnes of CO_2eq_ (Farm 8).

**Figure 4:**
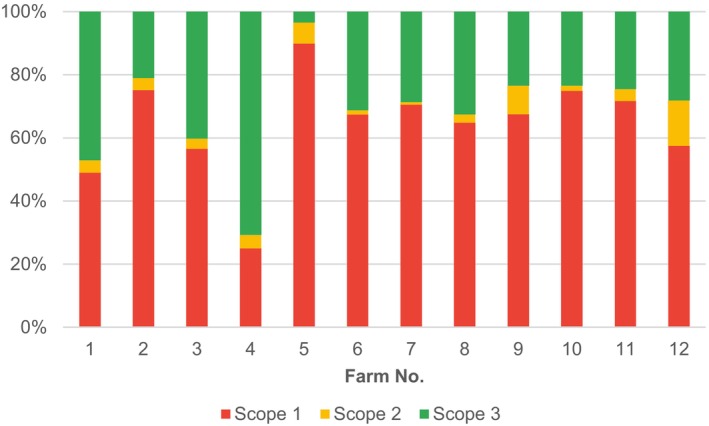
Share of Scopes in the farms’ total CFs for 12 tested farms, Czech Republic, 2022.

We assume that the complexity and focus of farm activities and production matter. Therefore, we first disaggregated total results (farms’ total CFs) by the CF Scopes (Fig. 4).

Scope 1 GHG emissions (i.e. direct emissions from activities that take place at farms) comprise over 50% of total CFs for all farms except for Farms 1 and 4, and the share is as high as 90% for Farm 5. Scope 3 indirect emissions from manufacturing inputs into the farms’ operation have the second highest share ranging from 4% (Farm 5) to 71% (Farm 4). Scope 2 indirect emissions from manufacturing electricity and heat consumed on farms have the lowest share, ranging from less than 1% (Farm 7) to 12% (Farm 12).

**Figure 5:**
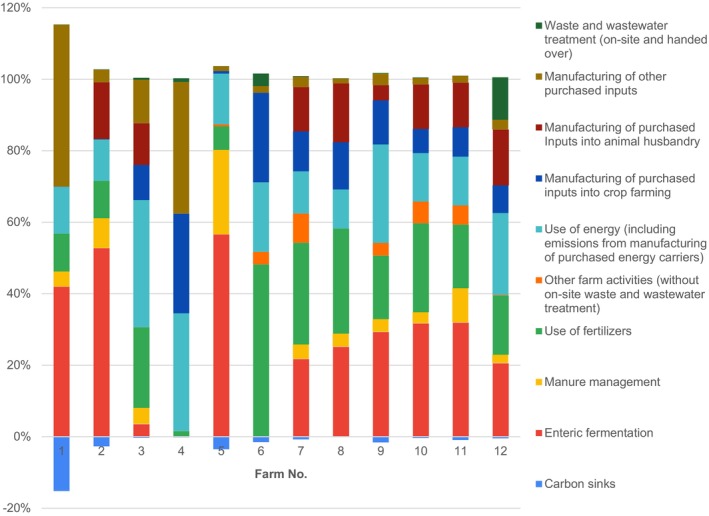
Share of particular GHG emission flows in farms’ total CFs for 12 tested farms, Czech Republic, 2022. The total share of all categories exceeds 100% as a result of the negative contribution of carbon sinks.

Second, we disaggregated farms' total CFs by particular GHG emission flows, as shown in Fig. 1, which allows for better insight into what drives CFs total results (Fig. 5).

Enteric fermentation has the highest share in total CFs with considerable variation, as high as 57% (Farm 5), although also zero for two farms with only crop production (Farms 4 and 6). Three other significant GHG emission flows include the use of fertilizers, the use of energy and the manufacturing of purchased inputs into crop farming. The use of fertilizers ranges from 2% (Farm 4) to 48% (Farm 6), the use of energy ranged from 11% (Farm 8) to 36% (Farm 3) and the manufacturing purchased inputs into crop farming ranges from zero (Farms 1 and 2) to 28% (Farm 4). The lowest shares were recorded for other farm activities (from zero for Farms 1–4 and 8 to 8% for Farm 7) and waste and wastewater treatment (less than 1% for most farms to 12% for Farm 12). Carbon sinks are the only GHG emission flows with negative shares, which decrease total CFs. They range from less than −1% (Farms 3, 4, 8 and 10) to −15% (Farm 1).

## DISCUSSION

### Data availability and uncertainties

The CF accounting system at the farm level was designed to minimize the amount of data input required from farms. Consequently, all tested farms were able to easily provide the necessary information for calculating Scope 1 direct emissions and sinks, as well as data on electricity and heat purchases, along with most other farm inputs. However, certain items, such as the inventory of machinery by type and information on waste and wastewater treatment, were not always readily available and required additional data collection.

The proposed accounting framework offers a comprehensive overview (a big picture), enabling the calculation of CFs for entire farms across all activities. Yet another option would be the CF assessment of specific products, such as a liter of milk or a kilogram of wheat, produced by different farms, or comparison CFs of various related products from a single farm (e.g. milk powder, milk, butter, cheese). To achieve this, it would be essential to organize the data entering the CF calculation by the produced commodities. Although some studies focus on the CFs of specific agricultural products,^18^, ^19^, ^20^ discussions with farm managers indicated that data related to fuel consumption by machinery and electricity usage is typically aggregated. Therefore, acquiring per‐product data regularly would necessitate the implementation of new data acquisition and management procedures at farms.

Both the data inputs and CF results are loaded with uncertainties. The Czech Hydrometeorological Institute^13^ quantifies these uncertainties for Scope 1 emissions, which are calculated using standardized equations from the IPCC Guidelines,^9^ with a range of 20–50%. Additionally, uncertainties associated with Scope 2 and 3 emissions may arise from the use of generic coefficients derived from the Ecoinvent database.^16^ To effectively manage these substantial uncertainties, it is essential to apply the proposed accounting framework consistently across all farms and, once available, to the entire time series. This approach would ensure that inter‐farm and inter‐year comparisons remain valid because it is likely that the uncertainties will exhibit similar magnitudes and directions across different farms and years.

### Benchmarking and assessment of the farms’ CFs

In general, regardless of whether we examine total emissions or emission intensities, there are three distinct approaches for interpreting farm CFs and identifying opportunities for reduction: (1) mutual comparison of CFs across different farms, (2) trend analysis of CF development over time and (3) evaluation of CF results against relevant reference values.^21^ Given that our CF calculations, conducted as a pilot analysis, focus on a single year and no reference values have been established, a comparative analysis emerges as the most applicable option for our case study.

The CF of agricultural farms can be assessed using benchmarking based on either absolute or relative values. This approach enables comparisons across different farms, helps identify best practices, and supports the setting of realistic reduction targets. Absolute CF measures the total GHG emissions produced by a farm, usually expressed in tons of CO_2eq_ per year. This metric provides a clear picture of the farm's overall environmental impact and is useful for tracking year‐over‐year progress in emission reductions. Relative CF, on the other hand, assesses emissions per unit of output, offering insights into carbon efficiency. Common metrics include emissions per turnover, employee and hectare of farmland, emissions per ton of product (crops or livestock), and emissions per calorie or protein unit, etc. These relative metrics are valuable for comparing production efficiency and allow meaningful comparisons between farms of different sizes and types.^22^

The comparison of absolute CFs shown in Fig. 2 is not particularly useful for benchmarking and assessment because of the size effect; larger farms are more likely to have higher absolute CFs than smaller farms. This assumption is supported by the Spearman correlation coefficients, which indicate a relationship between the farms' absolute CFs and factors such as turnover, area and number of employees. The values of these coefficients are 0.92, 0.98 and 0.95, respectively, which indicates a strong direct association between these variables.

In the case of relative CFs presented in Fig. 3, the effect is no longer at play, but another phenomenon appears there: a scale effect. A scale effect also referred to as economies of scale, is originally an economic concept assuming that larger economic subjects can do things more efficiently and have lower per‐unit fixed costs with the growing quantity of produced output.^23^ As shown, for example, by Arunrat *et al*.^24^ and Wang *et al*.,^25^ it can also be applied to GHG emissions from farming and suppose that larger farms will be more environmentally efficient and will have lower relative GHG emissions per various economic variables describing their size. To test the scale effect on our sample of farms, we calculated Spearman correlation coefficients between turnover and CF per turnover, area and CF per area, and number of employees and CF per employee. We assumed that, if the scale effect was the only factor responsible for the variability of relative CFs, the coefficients would be equal to −1 under the assumption of the linear relationship between the correlated variables. However, the calculated coefficients equaled −0.29, 0.48 and 0.06, respectively. The negative values for turnover and CF per turnover indicate some level of the scale effect but, because the coefficient is far from being close to −1, there are some other factors responsible for a major part of the variability of this relative CF. No scale effects were recorded for CF per area and CF per employee.

According to the literature, animal husbandry is much more GHG emission‐intensive than crop farming,^20^ which aligns with biochemical principles on nutrient and energy transformation efficiency between trophic levels.^26^ Because our results in Figs 3 and 5 support this, we tested the assumption that farms focusing on animal husbandry tend to have higher relative CFs. To do so, we calculated shares of animal husbandry (SoAH) in the farms’ operation as ratios of particular GHG emission flows, which could be unambiguously attributed to animal husbandry (i.e. emissions from enteric fermentation, manure management and manufacturing of purchased inputs into animal husbandry), to total CFs. We are aware that also other GHG emissions, such as those from energy use, partly stem from animal husbandry, but their attribution to it would require very detailed data on energy use, which are mostly not available at farms. Table 2 shows the calculated SoAH, which ranges from zero (Farms 4 and 6) to 0.8 (Farm 5).

**Table 2:** Shares of animal husbandry (SoAH) in the farms’ operation for 12 tested farms, Czech Republic, 2022

Farm no.	1	2	3	4	5	6	7	8	9	10	11	12
SoAH	0.46	0.77	0.20	0	0.80	0	0.38	0.45	0.37	0.47	0.54	0.39

As a next step, we calculated Spearman correlation coefficients between SoAH and CFs per turnover, CFs per area and CFs per employee, which resulted in values of 0.87, 0.70 and 0.44, respectively. The values of 0.87 and 0.70 indicate a strong correlation between shares of animal husbandry and CFs per turnover and CFs per area, which means that the focus on animal husbandry is an important factor influencing the variability of these two relative CFs. The correlation is moderate for CFs per employee, implying that also other important factors stand beyond its variability. In general, these factors can include the above‐mentioned scale effect and various management options, such as a soil tillage method, selection and method of application of fertilizers, selection of used energy sources, etc. The combined influence of all these factors on the variability of relative CFs would be determined by multivariate linear regression.^27^ Because our sample of farms is not large enough to apply this method, we present a simpler method of graphical evaluation of relative CFs below.

We argue that the farms with high SoAH can hardly decrease their relative CFs to the level of farms with zero or low SoAH. The reason is that GHG emissions from enteric formation and livestock feed are determined by livestock physiology and can be only marginally influenced by management measures. Even though there are novel approaches to decreasing methane emissions from enteric fermentation, such as adding methane‐reducing agents into fodder, they have not achieved full market maturity yet and still need further research.^28^ The greatest potential for reducing GHG emissions lies in improving manure management because emissions from liquid manure storage are an order of magnitude higher than those from other storage methods.^13^ However, with a few exceptions, the contribution of GHG emissions from manure management to total CFs is relatively low (Fig. 5).

**Figure 6:**
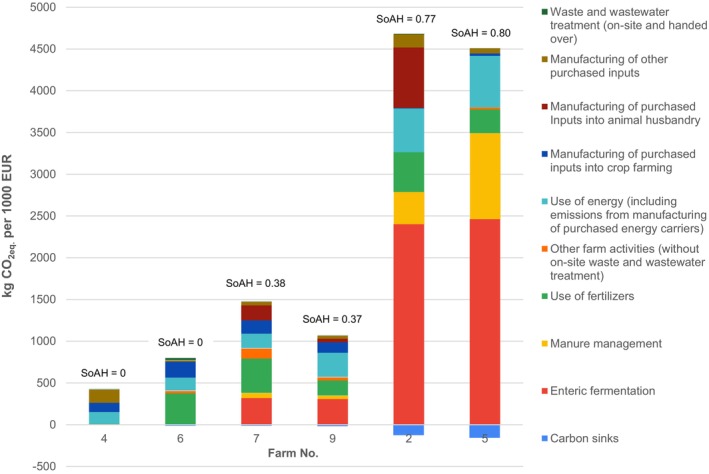
CFs per turnover broken down by particular GHG emission flows, for pairs of farms with similar SoAH, Czech Republic, 2022.

In this context, benchmarking and comparing different types of farms, with significantly different SoAH, is not particularly meaningful when identifying opportunities to reduce their relative CFs. To illustrate a more effective benchmarking‐based assessment, we present CFs per turnover, broken down by specific GHG emission sources, for pairs of farms with similar SoAH levels (zero, medium and high) in Fig. 6.

Farm 6 has almost twice as high CF per turnover as Farm 4. The major difference lies in GHG emissions from the use of fertilizers: Farm 4 grows grapes only and uses compost as a fertilizer, while Farm 6 grows many crops and applies large amounts of fertilizers, including industrial fertilizers, digestate, compost and crop residues. Although it cannot be expected that a conventional farm with a broad scope of activities would cut its fertilizer consumption to compost only, modern management practices such as precise farming might reduce its fertilizer consumption.^29^ A decrease in the consumption of industrial fertilizers would bring a double‐dividend, decreasing both GHG emissions from the use of fertilizers and emissions from the manufacturing purchased inputs into crop farming because emissions from manufacturing consumed industrial fertilizers compose 85% of this GHG emission flows. However, the comparison of Farms 4 and 6 also highlights the potential to decrease CF per turnover of Farm 4 as a result of its disproportional share of GHG emissions from manufacturing other purchased inputs at this farm. Half of this share comprises emissions from manufacturing construction materials, which might be a stand‐alone 1‐year purchase only. Still, the second half is related to the use of machinery at the farm. Although Farm 4 is almost 10 times smaller area‐wise than Farm 6, it uses twice as much agricultural machinery. This implies that there might be some potential for using this machinery more efficiently and decreasing its numbers.

Total CFs per turnover for Farm 7 is approximately 40% higher than for Farm 9. This farm has a comparatively much higher share of GHG emissions from manufacturing purchased inputs into animal husbandry, which is almost exclusively composed of emissions from animal feed manufacturing. This means that, if the farm attributed more of its agricultural land to fodder crops, the share of this category could be decreased. 60% of emissions from other farm activities stem from leakages of refrigerants. Farm 7 uses blended hydrofluorocarbon refrigerant known as R‐404A, which could be replaced with some refrigerant with lower or even zero global warming potential, such as ammonia.^30^ Last but not least, Farm 7 has a higher share of GHG emissions from fertilizer use. Similarly to Farm 6, Farm 7 uses a large amount of fertilizers, including industrial fertilizers, digestate and crop residues, and there could be a space to reduce this consumption by applying the principles of precise farming.

Farm 2 has approximately 5% higher total CF per turnover compared to Farm 5 and has a disproportionally high share of GHG emissions from manufacturing purchased inputs into animal husbandry. Unlike Farm 7, Farm 2 does not produce any crops for sale, and so its agricultural land is already fully used for farming fodder crops. The Farm should therefore, try to increase their yields through better land management, more efficient fertilization, different fodder crop selection and/or rotation, etc.^31^ On the other hand, Farm 5 has a disproportionally high share of GHG emissions from manure management, with 73% of its manure stored as a liquid. As mentioned above, GHG emissions from liquid storage of manure are an order of magnitude higher than other types of manure storage. This means that investments in facilities for solid storage of manure or even biogas stations would decrease the share of this category significantly.

All the farms shown in Fig. 6 also can seek to increase carbon sinks into the soil and thus decrease their total CFs by application of no‐tillage farming. Measurement of carbon sinks for no‐tillage farming has just started (worldwide, as well as in the Czech Republic) and provides ambiguous results. We used a conservative value of 60 kg of stored carbon in soil per hectare for no‐tillage farming in our methodology,^15^ and the modeling results showed that even this low value had the power to decrease total CFs by approximately 20%. Still, no farm in our 12‐farm sample has applied no‐tillage farming yet. As regards energy use, a feasible approach with respect to decreasing related GHG emissions is an investment in biogas stations and solar panels because electricity and heat from these facilities are less carbon intensive than electricity and heat from conventional sources.^16^ Out of the six farms in Fig. 6, no farm has solar panels, whereas Farms 6, 7 and 9 use their biogas stations.

The suggested measures for reducing the carbon footprints of farms are derived entirely from the collected data and their analysis. The next step will involve returning to the farms to present and explain the results, as well as to develop actionable steps that can transform these potential measures into practical actions. These actions, as summarized in Hermansen and Kristensen,^32^ must consider the economic, environmental and other specific conditions of each farm and, therefore, cannot be designed without their direct involvement. Although this follow‐up is planned, we consider it beyond the scope of the present study.

## CONCLUSIONS

We have developed a framework for CF accounting at the farm level and tested it on 12 farms in the Czech Republic. Our findings indicate that the data availability at these farms is sufficient in terms of quality and quantity to routinely conduct whole‐farm CF calculations. However, the available data are insufficient for calculating CFs for individual farm products, such as milk, wheat or meat. Such product‐specific calculations would require the establishment of new data acquisition and management procedures at the farm level.

A key aspect of our research was to examine how different farm characteristics and other factors influence the overall CF and how farms can be benchmarked based on their CFs to provide valuable insights for relevant stakeholders. We found that absolute farm CFs are significantly influenced by the size effect, making them unsuitable for benchmarking purposes. By contrast, relative farm CFs (per turnover, per area and per employee) are not affected by the size effect, although a scale effect was observed for CFs per turnover only.

Additionally, we investigated whether a focus on animal husbandry leads to higher relative CFs, aligning with existing literature and our results in Figs 3 and 5. By calculating the SoAH in farm operations, we discovered a significant correlation between SoAH and relative CFs, with the strongest correlation observed for CF per turnover (0.87). Despite this high correlation, other factors also contribute to the variability of relative CFs, especially for CFs per area and per employee, which showed lower correlations of 0.70 and 0.44, respectively. These additional factors may include scale effects and various management practices, such as soil tillage methods, fertilizer selection and application, and types of energy sources used.

We argue that farms with high SoAH are unlikely to reduce their relative CFs to the levels of farms with zero or low SoAH. This is because GHG emissions from animal husbandry are largely determined by livestock physiology and can only be marginally influenced by management practices. Therefore, benchmarking and assessing farms with significantly different SoAH levels is not particularly meaningful when identifying potential strategies to reduce relative CFs. Instead, we propose applying benchmarking to farms with similar SoAH, as demonstrated in Fig. 6.

Once CF data become available for a larger number of Czech farms, it will be possible to establish reference values based on statistics, such as the average shares and average absolute values of specific GHG emission flows that contribute to total CFs at various levels of SoAH. This will enable the assessment whether farms’ total CFs and their components are above or below average, as well as the assessment of the distance between individual farms and the best‐performing values, highlighting potential areas for CF reduction. These reference values will likely be country‐specific for the Czech Republic because the environmental conditions and institutional frameworks relevant to agriculture may differ from those in other countries. Additionally, a key challenge will be the application of farm CF results to establish ‘sustainable reference values’ that consider both scientific and political criteria and concerns.

## Supporting information


**Data S1.** Supplementary Information.

## Data Availability

The data that support the findings of this study are available from the corresponding author upon reasonable request.
